# Learning through story: how peer-led support groups use storytelling to empower adolescents living with HIV in Côte d’Ivoire

**DOI:** 10.1186/s12913-026-14944-x

**Published:** 2026-06-23

**Authors:** Marc Kouadio N’goran, Leila Katirayi, Godfrey Woelk, Ashleigh Hayward, Randi Holmes, Ignace Tosseu Ban, Martha Mukaminega, Carlos E. Rodriguez-Diaz

**Affiliations:** 1 Elizabeth Glaser Pediatric AIDS Foundation, Abidjan, Côte d’Ivoire; 2 Elizabeth Glaser Pediatric AIDS Foundation, Washington, D.C. USA; 3 George Washington University, Milken Institute of Public Health, Washington, D.C. USA; 4 Boston University School of Public Health, Boston, MA USA

**Keywords:** Adolescent, HIV, Support group, Peer support, Storytelling, Cote d’Ivoire

## Abstract

**Background:**

Adolescents living with HIV struggle to accept their HIV status and remain adherent to antiretroviral therapy (ART). Support groups are one way to assist adolescents overcome these barriers. This evaluation, conducted in health facilities in Abidjan, Côte d’Ivoire, explored the acceptability of peer-led support groups using a ten-topic curriculum and a ‘storytelling’ model in which fictional or real-life scenarios were used to make information more relatable and encourage discussion and sharing of personal experiences.

**Methods:**

In-depth interviews were conducted to explore the acceptability of the new model among adolescents enrolled in peer-led HIV support groups, peer support group leaders, and health care workers. The study also sought to describe the experiences of peer leaders and HCWs involved in implementing the latest model. Data were collected on the percentage of adolescents who created treatment plans, and session and topic attendance, from six facilities providing peer-led support groups. Interviews were audio-recorded, transcribed, and translated. Data were approached from a constructivist perspective, and thematic analysis was used to identify themes within participants’ accounts. A code list was created using a combined inductive and deductive approach. Transcripts were coded using MAXQDA v.12.

**Results:**

A total of 45 in-depth interviews were conducted from August to September 2021. Across participant groups, peer-led support groups were perceived as highly important sources of support for adolescents living with HIV. Adolescents reported increased comfort in sharing their personal experiences and engaging with content delivered through storytelling. Participants described the development of trust and peer support within groups, while peer leaders were viewed as role models for medication adherence. Health care workers reported improved communication and relationships with their adolescent patients. Key barriers to participation included scheduling constraints and transport costs Participants attended an average of six of the ten sessions, with the highest attendance observed for sessions on safe sex and disclosure of HIV status.

**Conclusion:**

Peer-led support groups provide critical support to adolescents in accepting their HIV status. The support groups helped adolescents to learn the importance of ART adherence and create a supportive environment for them to live positively with HIV.

**Supplementary Information:**

The online version contains supplementary material available at https://doi.org/10.1186/s12913-026-14944-x.

## Background

Sub-Saharan Africa remains an epicenter of the HIV epidemic. Although HIV prevalence in West and Central Africa is lower than in other regions of Sub-Saharan Africa. Côte d’Ivoire (CDI) continues to have one of the highest HIV prevalence rates in West Africa. According to the 2024 UNAIDS estimates, approximately 410,000 people are living with HIV in CDI, with an HIV prevalence of approximately 1.7% among adults aged 15–49 years [1]. Women remain disproportionately affected, with HIV prevalence estimated at 2.4% compared to 1.0% among men aged 15–49 years [1]. While progress is being achieved in adolescent HIV care, there are still significant gaps in the healthcare system and a lack of services for this population, including integration of HIV testing in all settings and retention in treatment [2].

Adolescents living with HIV (ALHIV) face unique challenges compared to adults. They can experience complex emotional and behavioral problems during this vulnerable transition period [3]. Psychosocial challenges, such as experiencing HIV stigma from both peers and the community, have been found in multiple studies as a primary stressor in ALHIV [4–6]. Among ALHIV, the fear of gossip and losing friendships if their HIV status was discovered in their social circles has been documented as a concern [4]. Because of the discrimination ALHIV face, their mental health is often affected with a significant decline in self-esteem, and they also have a high risk of depression and anxiety [5]. A challenge less documented is the educational impact of missing multiple school days due to appointments or periods of severe illness. This can result in falling behind in their education without the opportunity to catch up with their classmates and repeating the same grade level [6]. Additionally, it can be challenging to maintain adherence during school, as many ALHIV hide their medication to avoid disclosing their status to peers [4, 6]. Poor ART adherence is multifactorial, including psychosocial challenges, developmental challenges, stigma/disclosure concerns, transition-related disruptions and structural barriers to care [7]. 

### Peer-led adolescent support groups

Support groups are commonly offered for people with HIV [8]. However, for ALHIV, these support groups may be even more critical, as they are in a stage of their life where peer influences are crucial in adolescent development [9]. Adolescents face many challenges, including accepting their HIV status and navigating social and peer circles with their HIV status [9, 10]. Support groups are one of the approaches to help adolescents address these challenges.

The majority of adolescent support group models in CDI at Elizabeth Glaser Pediatric AIDS Foundation (EGPAF) supported sites are led by health care workers (HCWs) or lay counselors. Previous support group models allowed adolescents who did not know their HIV status to enroll, which somewhat limited the conversations. These previous support groups permitted continuous enrollment, with new adolescents joining the support groups at any time. These support groups had limitations: adolescents were less likely to discuss their individual situations in the presence of adults, and participation and rapport were reduced when new adolescents were allowed to join at any time.

To address these challenges experienced at EGPAF-supported facilities, strengthen adherence to care and treatment services, and reduce stigma, EGPAF implemented a new peer-led support group model using a storytelling model for adolescents in CDI in 2020. The model was created from the recognition that there was limited engagement and consideration of adolescents’ voices in HIV programs and limited peer-led service and support groups. The intention was to improve peer care and support, and clinical outcomes for adolescents on treatment. The story-based model was used to stimulate discussion in a safe environment, with the belief that stories can lead people to change by creating emotional investment, capturing attention and interest, providing energizing messages, and motivating individuals to act. For each story, there was a character that was like the participants (age, HIV experience, etc.), a situation that was realistic, a challenge/opportunity, a response that models possible actions, and an outcome. The story always ended positively, even if it started negatively.

The support groups were in six pediatric and adolescent high-volume sites, including three university teaching hospitals, one district hospital, and two community-based health centers. A high-volume site is classified by the Ministry of Health and implementing partners as a facility with more than 200 adolescents and children actively on care. Adolescents in the support groups were between the ages of 15–19. Support groups were led by HIV-positive young adults 19–24 years and older who were adherent on ART. They were trained to be ‘peer leaders,’ and act as role models for adolescents in the group. The peer leaders were trained in support group conduct and management. Peer-leaders received transport reimbursement for the days they supported sessions; no other financial support was provided for other participant groups. A 3-day training was held, and then a 5-day practical phase was conducted, where HIV-positive adolescents were assembled to form a ‘pilot’ support group for the peer-leaders to gain the experience of leading a group.

This new model used ten pre-determined topics (see Table 1). Key messages were provided for each of the ten topics. A story accompanied each topic to spark an engaging conversation in the group, encouraging adolescents to feel comfortable in sharing their own experiences and learn from each other. Peer leaders were also encouraged to use their own stories to lead the conversations as well. The storytelling strategy involves sharing a story of a fictional (or sometimes real) character that is facing the challenges of the selected topic. The story is shared by the peer-leader and the participants in the group are asked to comment on how the fictional character handled the situation, what could have been done differently, or done better. This model prompts adolescents to discuss real-life situations and how they have handled them. The groups met weekly, either on the weekends or sometimes on Wednesday afternoons when school was not in session. The groups could continue to meet at the facility after the ten sessions were completed, but this was done informally and at the discretion of the individual groups.

The new peer-led model allowed adolescents to join the support group only at its formation. Once at least 15 adolescents joined the group, it was closed, and new adolescents joined the next group. The peer leaders reported that closing entry into the group allowed adolescents to develop trust and form friendships.

The new support groups were led by peer-leaders aged 19–24 who were adherent to their ARV treatment, and by a support group supervisor, typically the pediatric social support focal person at the site (a social worker). At some of the sites, the social support focal person would attend the support groups monthly (or at request) to ensure the program was running smoothly; individual sites decided this. HCWs, referring to different positions employed at the facility that supported the support groups, such as social workers, community advisors and doctors, were also available for any medical questions and any functional support needed to run the groups. This care team, the peer-leader, support group supervisor, and HCWs worked directly with the adolescents to create individual treatment plans in which each adolescent outlined their treatment goals, identified potential challenges they may encounter, and developed appropriate, advance-planned solutions. The treatment plan was introduced with the new peer-support group model. The plan was created with the adolescent, peer leader, support group supervisor, and the lay counselor within one month of the adolescent joining the group and was revisited after completing the 10 sessions. Unlike the previous model, in this model, adolescents were required to know their HIV status, making these treatment plans possible.

In summary, the main differences between the old support group model and the new model were that in the new model the peer leaders led the support groups instead of HCWs, a storytelling model was used to prompt adolescents to discuss real-life situations and how to handle challenges they encounter, the new model only included adolescents who knew their HIV status, and that entry into the support group closed after the first session, ensuring more trust among the group.

**Table 1:** Topics (in the order taught)

Session Topic	Content
Accepting HIV diagnosis, self-care etc.	Explain the normal stages of learning HIV positivity to accepting HIV diagnosis for adolescents; highlight successful problem-solving that responds to self-identified needs (emotional, physical, and social)
Positive living	Appreciate commitments and accomplishments to live life with hope, dignity, and purpose
Disclosure (sharing HIV status)	Share disclosure experience and openly discuss questions and concerns
ART adherence	Raise the reality of lifetime adherence and the role of treatment supporters
Follow-up at the health center (including viral load monitoring)	Activities at follow-up health center visits; clarify the process and schedule of viral load tests and their meaning through ups and downs
Rights and stigma	Identify a time when stigma took away the rights of ALHIV
AIDS-free living	Describe a time when an ALHIV decided to take charge of his/her life with HIV and was determined to live AIDS-free
Safe Sex	Highlight preparing for sexual activity and using condoms to prevent HIV and pregnancy in sexual relationships
Achieving your personal goals and ambitions	As determined by group attendees and core members’ needs.
Transition to adult (care and life)	Share a transition from pediatric to adult care, acknowledging differences and patient responsibilities

## Methods

### Study design

The study was guided by the following research questions: (1) How do adolescents at selected sites describe their health-seeking behaviors and perceive the acceptability of the peer-led support model? (2) What are the experiences of adolescent peer educators and health care workers in delivering adolescent services at these sites?

This study was informed by a constructivist framework. A constructivist approach assumes that reality is socially constructed and seeks to understand how individuals interpret and make meaning of their experiences within specific contexts [11]. Qualitative data were collected through in-depth interviews (IDIs), and quantitative data were collected on attendance at support group sessions, the creation of individual treatment plans, and preferred meeting topics. In addition to exploring the acceptability of the new model among HIV-positive adolescents, the study also sought to describe the experiences of peer leaders, and HCWs involved in implementing the latest model.

### Site selection

Of the 15 sites that implemented the peer-support groups using the storytelling model, six health care facilities located in Abidjan were selected as study sites. The sites were purposively selected due to their high number of adolescents accessing HIV services. The sites were urban with all populations welcome, including wealthy, poor, and migrants.

### Study population and eligibility

In-depth interviews (IDIs) were conducted with ALHIV enrolled in the support groups, peer leaders, and HCWs. Adolescents were eligible for an interview if they were 15–19 years old, on ART, attended one of the study health facilities, were enrolled in a peer support group and attended at least 5 sessions. Participants were limited to those who attended at least five of the ten sessions, so that they had more experience in the support groups. Peer leaders were eligible to participate if they had held the peer leader role for at least 3 months and supported adolescents in developing treatment plans. HCWs were eligible if they worked in a study health facility and were involved in HIV care and the treatment services for adolescents and implementation of the peer-led support groups for at least six months.

### Recruitment & sample size

Interviews were conducted after all ten sessions of the support group had been completed. Adolescents were informed about the study by peer leaders during the support group meeting and invited to join the study using volunteer selection. If more than two adolescents of each gender wished to participate, the peer leader selected participants using a lottery system, drawing names from a bag. Participation in the peer-led support groups was independent from participation in the research study. Adolescents were informed that declining to participate in the interviews would not affect their access to support groups, clinical care, or relationships with peer leaders or HCWs. Peer leaders only introduced the study and obtained permission to share contact information with the research team; all consent and assent procedures were conducted directly by research assistants (RAs). Participation in the interviews was entirely voluntary.

Sites had 1–2 peer leaders and 1–2 HCWs supporting adolescents. Where more than two peer leaders or HCWs were available, purposive selection was used to identify the two with the most experience working with adolescents to participate. If there were fewer than two adolescents, one peer leader, or one HCW available at the selected site, additional participants were recruited from another site.

Sample size was determined a priori based on established qualitative research conventions regarding thematic saturation. In applied public health research, particularly in multi-site studies, it is common to use evidence-based estimates of the number of interviews required to reach saturation rather than assessing saturation iteratively after each interview. Previous literature has demonstrated that saturation can be reached at 10–12 IDIs per homogeneous group; this informed the minimum sample size for each study population group in this study [12]. Consistent with this approach, sampling continued until the target sample sizes for each group were reached, which was considered sufficient to capture thematic redundancy across participants.

### Qualitative data collection

Data were collected from August-September 2021 by RAs hired as short-term staff for the study. The RAs were both male and female, from Abidjan, familiar with the local context and all had previous experience collecting qualitative data. They were trained in study procedures, data collection, qualitative interviewing techniques, ethical research practices and maintaining neutrality during data collection. Training included role-playing exercises using the interview guide under supervision of the lead trainer.

The interview guides included questions about the perceptions and experiences of the peer-led support group using the storytelling method, perceived changes to ART adherence, adolescent challenges with treatment, and recommendations for improving the peer-support model. The interview guides were developed specifically for this study (see supplementary material) and were developed in English, translated to French and then back translated for quality control.

Written informed consent was obtained from all participants before data collection. For adolescents under 18 years of age, parents or guardians provided written consent, and adolescents provided assent. Participation in the peer-led support groups was independent from participation in the research study. Adolescents were informed that declining participation in the interviews would not affect their access to the support groups or clinical care. All interviews were conducted in French at the health facilities, lasted approximately 30–60 min, and were audio-recorded with only the participant and RA present. Interviews were conducted using a standardized guide, the English version of which is included in Appendix A. RAs also generated field notes documenting observations, reflections, and contextual details during or after interviews. No financial incentives were offered, no participants declined participation or withdrew from the study, and no repeat interviews were conducted.

No relationships had been established between the RAs and participants prior to data collection. RAs introduced themselves and explained their independent role in the study before each interview. Because participants may still have perceived RAs as affiliated with the health system or support program, RAs emphasized confidentiality and encouraged honest feedback, including both positive and negative experiences.

The broader study team included individuals with technical expertise in HIV and adolescent programming, which informed study design and interpretation. To support reflexivity, the analytical team held regular discussions during analysis to reflect on how their professional backgrounds and assumptions could shape the interpretation of the data and to ensure findings remained grounded in participants’ perspectives.

### Quantitative data collection

Quantitative data were collected from all adolescents enrolled in the support groups, also from August-September 2021. These data were collected by RAs, who abstracted information from registers at the health facility and entered it into electronic tablets for study purposes. These data included demographics, the number of adolescents with a treatment plan, support group meeting attendance, and attendance levels for the different topics.

### Data analysis

Audio recordings of the interviews were transcribed verbatim into French by the same RAs who conducted them. To ensure transcription quality, RAs reviewed transcripts against the audio recordings for accuracy and completeness. The French transcripts were then translated into English by two bilingual RAs fluent in both French and English. To maintain translation quality, a subset of transcripts was reviewed by a second team member to check for consistency and accuracy of meaning between the French and English versions. Transcripts were not returned to participants for comments or correction.

Local vernacular and colloquial expressions were retained in the original French transcripts and translated into English using phrasing that best reflected the intended meaning and cultural context. Where direct translation was not possible, translators used equivalent expressions and, when necessary, included clarifying language to preserve the nuance of participants’ responses.

Two RAs, under the supervision of a lead qualitative scientist (LK), generated a code list using a combined inductive and deductive approach. The code list was developed through an iterative process combining inductive and deductive coding approaches. Initial codes were generated from repeated readings of the transcripts and informed by the study objectives and interview guides. Similar codes were grouped into broader categories and subcategories through team discussion and refinement. The final code list included major thematic domains related to participant experiences, barriers and facilitators to participation, perceptions of storytelling, psychosocial support, and implementation considerations. The coding framework was continuously refined throughout analysis to incorporate emerging concepts and relationships across participant groups. Transcripts were coded by two RAs in the qualitative analysis software, MAXQDA v. 12. Study participants were not engaged to provide feedback on the findings.

All audio recordings and transcripts were stored on secure, password-protected servers accessible only to authorized study team members. Transcripts were de-identified prior to analysis, and unique study IDs were used in place of participant names. Data management and storage procedures were overseen by the in-country PI (MKN), with RAs responsible for transcription, translation, and data organization.

To support analytic rigor and trustworthiness, a subset of transcripts was double coded by two RAs, with discrepancies discussed and resolved through consensus. Regular meetings were held with the lead qualitative scientist to review coding decisions, refine the codebook, and ensure consistency in code application.

Once coding was complete, code reports were generated for each code by study population. These reports were used to summarize the data through descriptive, text-based summaries and data display matrices (data reduction tables). Data were organized by study population groups (participants, peer leaders, and HCWs), allowing comparison of similarities and differences across groups. Related codes were grouped into overarching themes, and illustrative quotes were selected to support thematic findings. Quantitative data were summarized using frequencies, medians, means, and proportions.

Once coding was complete, code reports were generated for each code by study population. These code reports were used to then summarize the data through descriptive, text-based summaries and data display matrices (data reduction tables). Data were organized in these tables by study population groups (participants, peer leaders, and HCWs) to compare similarities and differences across groups. Related codes were grouped into overarching themes and illustrative were selected to support thematic findings. The quantitative data were summarized using frequencies, medians, means and proportions.

## Results

Across the six sites, 21 IDIs were conducted with adolescents who participated in the support groups, 12 IDIs were conducted with peer leaders, and 12 IDIs were conducted with HCWs, for a total of 45 IDIs.

During the data collection period, August-September 2021, approximately 116 adolescents were enrolled in the peer-led adolescent support groups. Demographic data for this group are presented below, compared with those of the 21 participants in the IDIs. The median age and (interquartile range, [IQR]) of the adolescents was 16 (15;17) years, and 53% (*n* = 61) were male (Table 2). Of the 116 adolescents who participated in the support groups, 104 reported their education level: 16% (*n* = 15) had no education, 33% (*n* = 33) had primary education, and 51% (*n* = 51) had secondary or higher education. The adolescents who participated in the IDIs were slightly older than the typical support group member (17 vs. 16) and more educated, with all interviewed participants having secondary or higher education.

**Table 2:** Demographic characteristics of support group participants

	IDI participants *N* = 21 *n* (%)	All peer-led support group participants* N = 116 *n* (%)
Median age in years (IQR)	17 (16,19)	16 (15,17)
Sex		
Male	13 (61.9)	61 (52.6)
Female	8 (38.1)	55 (47.4)
Education	(*N* = 21)	(*N* = 104)
None	0	15 (15.6)
Primary	0	38 (33.3)
Secondary or higher	21 (100)	51 (51.1)

*The 21 adolescents who participated in the IDIs are included in this group of 116 adolescents

A total of twelve peer leaders participated in the IDIs, six male and six female, with a median age of 21.5 years (IQR: 19–22). All peer leaders were single, had no children, and had at least a secondary education. Of the 12 HCWs were interviewed; all were female and had an education level of secondary or higher. The types of HCWs interviewed included social workers, doctors, community advisors, and clinical advisors. Years of work experience ranged from less than a year to 17 years.

### Qualitative findings

The analysis focused on the use of the storytelling model, exploring attitudes towards the storytelling model, how it shifted experiences living with HIV and taking treatment, and perceived behavior changes. Data were analyzed among the three study populations, adolescents, peer leaders, and HCWs. Results have been presented across three main themes, including connection & resonance, storytelling & change, and challenges and sustainability (see Table 3). Within these overarching themes, sub-themes have been identified. Selected quotes have been provided to give voice to the participants’ unique experiences. Some quotes are used to represent more than one sub-theme.

**Table 3:** Overview of themes related to storytelling in qualitative data

CONNECTION & RESONANCE				
Thematic Domain	Adolescents	Peer Leaders	Health Care Workers	Comparative Interpretation
Identification and Connection	Adolescents consistently said they saw themselves in the stories and felt concerned or ‘in the person’s shoes.’ Stories mirrored their own realities (school, stigma, treatment). *It’s a good idea because thanks to these stories*,* each of us feels ourselves in them. There is a story that impacts every person.* Male, 18, 12A04	Peer leaders used stories and their own testimonies to help others identify with shared struggles. *I used my personal story during the first session — I said how old I was when I got the disease and what I went through. They listened and opened up too.* Female, 19, 15P02	HCWs observed that stories helped youth relate to content. *It motivates the children even more because we tell them that these are real stories edited by their friends.* Female, Social worker, 14H02	Consensus: All groups viewed identification as the core mechanism of storytelling’s impact. Adolescents experienced it personally; peer leaders intentionally used it as a facilitation tool; HCWs recognized it as the method’s foundation.
Authenticity and Credibility	Real stories (not fictionalized) felt trustworthy. *The stories are real; they’re not invented. That’s why we listen.* Female, 16, 11A01	Peer leaders emphasized authenticity through personal disclosure. *Our real stories make them believe it*. Female, 19, 15P02	HCWs confirmed authenticity as essential. *Everyone finds themselves in these stories. That’s when they examine their conscience and say*,* really what I have [HIV]*,* I have to take it seriously.* Female, Social worker, 14H02	Consensus: Authenticity = credibility = engagement. Difference: Adolescents valued realism; peer leaders embodied it; HCWs validated its pedagogical power.
Emotional Resonance and Empathy	Stories touched, moved, or motivated adolescents. An emotional connection often leads to behavioral change. *When I was listening to that story*,* I identified heavily with that girl*,* and it made me feel good.* Female, 16 12A02	Peer leaders intentionally leveraged emotion- they noticed when participants cried, reflected, or felt encouraged. *We ask for the feeling after a story. Some will say they did not feel anything. Others will say what part they liked or didn’t like.* Female, 19, 14P02	HCWs saw emotion as both positive (deep engagement) and challenging (required containment). *It’s easier to touch the heart with a story than with advice… Sometimes it makes them emotional. When they see themselves in the story*,* they cry*,* and we must comfort them*. Female, Clinical advisor, 11H02	Consensus: Emotion drives change. Difference: Adolescents experienced it; peer leaders managed it; HCWs moderated and contained it professionally.
**STORYTELLING & CHANGE**				
Comprehension and Learning	Stories made abstract messages concrete. *When the stories are told*,* I feel myself in them because I experienced the same… it helps me know how to take care of myself.* Male, 17, 12A01 *Peers are even better because we understand each other. We’re the same*. Male, 17, A1603	*It teaches us and it informs us about things we had no idea it could be. It educates us*,* in a way* Male, 21, 13P01	HCWs echoed this, contrasting storytelling with ‘lectures’ and noting improved engagement and retention. *Stories make things more concrete. Before*,* we gave health talks*,* but the children were distracted. Now they are attentive. They say*,* ‘It’s not theory; it’s real.’ They react; they ask questions. * Female, Community advisor, 12H03	**Consensus**: Storytelling improved comprehension across all groups. **Difference**: Adolescents described intuitive understanding; peer leaders described intentional teaching design; HCWs observed measurable comprehension gains.
Facilitation, Structure, and Pedagogy	Adolescents generally reported appreciating the storytelling model. *The stories… make it easier to understand the message well* Male, 15, 14A03 .	After reading the story, there is the section ‘let’s think together.’ *After we read the stories there is application and practice*,* where we discuss the advice of the story in our own daily lives.* Male, 23, 15P01	HCWs recognized that adolescents were more comfortable and engaged with this new model. *I can say that when it’s a health worker*,* it’s like a court*,* like in school. But when it’s a peer*,* they are even more comfortable.* Female, Social Worker, 16H01. *They answer questions. In any case*,* they participate a lot.* Female, Clinical Advisor, 16H02.	**Consensus**: Structured storytelling improved participation. Difference: Adolescents perceived engagement; peer leaders practiced methodology; HCWs evaluated its instructional quality.
Behavioral and Clinical Impact	Adolescents reported concrete behavior changes: improved adherence, hope, openness. *A peer leader’s story inspired me—he was once very sick*,* now looks healthy. That encouraged me to take medication. These stories made me believe in my future and not give up.* Male, 18, 13A04 *I went from detectable to undetectable after the stories.* Male, 17, 14A02	*There was one last one who in any case was not taking his medication because he was out all the time… we told him to put an alarm on his phone… and that’s what he did* Male, 22, 16P01	HCWs perceived improvements in adherence, more consistent appointments, and viral load suppression among some adolescents. *We’ve seen children who didn’t take medicine before*,* now taking it regularly because of the stories and the peer leaders*. Male, Doctor,12H02	**Consensus**: All groups linked storytelling to positive behavioral and clinical outcomes. Difference: Adolescents described change internally; peer leaders witnessed it socially; HCWs measured it clinically.
Self-Acceptance of One’s HIV Status	Adolescents discussed the challenges of not only accepting one’s HIV status but the acceptance of taking daily drugs. *I had already accepted it*,* but the fact that I confided in others and that there were others in the same situation made me more confident and easier to accept.* Male, 20, 16A01 *This topic has helped me to understand that HIV is now part of my daily life and that I can have a normal life despite it*. Female, 18, 16A04.	Peer leaders used their own stories about learning their HIV status and coming to terms with it. *I used my personal story during the first session — I said how old I was when I got the disease and what I went through. They listened and opened up too. Some of them didn’t know that you can live a long time with HIV. The story*,* and my story*,* helped them see that.* Female, 19, 15P02	Through relatable, emotional, and hopeful narratives, storytelling fostered understanding, reassurance, and personal acceptance. E*veryone finds themselves in these stories… they examine their conscience and say*,* really what I have*,* I have to take it seriously* Female, Social Worker, 14H02	**Consensus**: Storytelling created safe, non-threatening spaces to come to terms with one’s HIV status. **Difference**: Adolescents experienced it as protection; peer leaders used it as a facilitation strategy; HCWs saw it as a therapeutic mechanism.
***CHALLENGES & SUSTAINABILITY***				
Emotional and Logistical Challenges	A few adolescents noted repetitive or long stories; some found parts were sensitive. *Some stories made me sad*,* especially when someone was sick or rejected. * Male, 18, 11A03 Adolescents also discussed challenges with transportation to be able to attend the support group sessions. *There are times when I don’t even have money for transport to come.* Male, 20, 16A01	Peer leaders mentioned shyness, difficulty managing emotions, and occasional repetition. *“We need more training to know how to help when they start to cry” * Male, 23, 15P01 Peer leaders also discussed wanting more support *We do our best*,* but sometimes we feel forgotten. We need more meetings with the staff to plan together.* Male, 22, 16P02	HCWs discussed concerns regarding insufficient training time for peer leaders and that more sessions being needed for adolescents to fully grasp the content. *The difference is that children were not coming to the group discussion for lack of transport.* Female, Community Advisor, 15H02 *The support group was a bit too fast. They didn’t retain everything because there wasn’t enough time to go deeper into the stories*. Female, Social Worker, 16H01 *Now*,* we have meetings outside where everyone comes to see the themes on the board.* Female, Community Advisor, 15H02	**Consensus**: All groups saw minor challenges. **Difference**: Adolescents focused on content; peer leaders on facilitation hurdles; HCWs on implementation and structure.
Perceived Value and Sustainability	Adolescents overwhelmingly praised storytelling as motivating, fun, and helpful. *It’s interesting. We learn and we laugh too. It’s not boring like before. * Male, 17, 11A02	Peer leaders called it transformative, authentic, and ‘easy to use.’ *The stories are simple*,* and they make it easier to understand the themes* [topic areas]. Female, 19, 12P01	HCWs viewed it as effective, participatory, and worth continuing, recommending updates and multimedia integration. *The stories help because they show models of success. They see the model in front of them and realize that the person has done well. So they do the same thing. We can improve it with visual tools*,* electronic tools because we are in the electronic age.* Female, Doctor, 14H01	**Consensus**: All judged the method highly effective. **Difference**: Adolescents valued experience; peer leaders valued empowerment; HCWs valued outcomes and sustainability.

### Quantitative findings

For the quantitative results, data were gathered on the percentage of adolescents in the support groups who created treatment plans, attended the ten sessions, and attended by meeting topic. These quantitative indicators were intended to describe program implementation and participation rather than measure clinical outcomes such as viral suppression, retention in care, or ART adherence.

As discussed earlier, each adolescent in the peer-led support groups created a treatment plan with their peer support leader and lay counselor. These treatment plans helped identify potential barriers each adolescent may experience in staying on treatment and brainstorm personal solutions to address them. Of the 116 adolescents enrolled in the new support group model, 112 had treatment plans (96.6%).

By the time of data collection, each peer support group had the chance to conduct 10 sessions. However, the average number of support group sessions attended by adolescents was six sessions.

We explored the attendance by topic. Table 4 presents the number and percent of adolescents who attended each topic of the 116 adolescents. Participants were informed in advance of the topics to be addressed. Before starting the program, the peer leaders and the facilitators presented the overview of the program, including the topics and order.

**Table 4:** Number and % of participants (attendance) per topic of support groups sessions (topics are in order presented

Session Topic	# of participants (%)
Accepting HIV diagnosis, self-care etc.	60 (51.7)
Positive living	65 (56.0)
Disclosure (sharing HIV status)	81 (69.8)
ART adherence	61 (52.6)
Follow-up at the health center	60 (51.7)
Rights and stigma	70 (60.3)
AIDS-free living	62 (53.4)
Safe Sex	81 (69.8)
Achieving your personal goals and ambitions	76 (65.5)
Transition to adult (care and life)	75 (64.7)

Among the **10** topics addressed during support group sessions, two of them, “Status sharing” and “Safe sex activity,” registered the highest attendances with 81 participants out of 116 **(69.8% of participation).** However, two other topics, “Accepting your HIV status” and “Follow-up at the health center,” were less attended by the adolescents. Only 60 of 116 participants (51.7%) attended both topics.

## Discussion

Most participants attended an average of six sessions. The most attended sessions covered the topics of “Safe sex” and “Sharing HIV status.” The preference for these topics is consistent with previous research findings demonstrating that ALHIV experience HIV stigma and discrimination [13] and difficulties with HIV disclosure. Attendance could be related to the transportation challenges that adolescents noted.

The results from the qualitative data have been grouped into three themes, including connection & resonance, storytelling & change, peer belonging, and disclosure.

### Connection & resonance

One of the aspects most valued by adolescents in our study was the sense of connection fostered by peer-led support groups and the extent to which the stories resonated with their lived experiences. Participants consistently emphasized how the storytelling model enabled them to identify with the characters and visualize their own circumstances, making the messages more meaningful and relevant. This is consistent with evidence that patient stories can enhance health education by increasing personal relevance and emotional engagement, thereby improving openness to and acceptance of information [14].

The design of the new support group model, which prohibited new members from joining after the first meeting, further strengthened group cohesion and trust. This structure aligns with prior findings showing that changes in group membership can undermine rapport and create anxiety among existing members who have already established friendships and mutual understanding (15).

Establishing social connections and community is particularly critical for adolescents living with HIV. Prior studies describe how adolescents often experience social judgment and stigma, reporting feelings of being “isolated and dehumanized outside the clinic” [15]. Similarly, adolescents in both our study and others described support groups as spaces of freedom and safety, where they could share openly without fear of discrimination. The ability to speak freely and build supportive peer relationships was repeatedly cited as one of the most meaningful benefits of the intervention.

Peer leaders played an essential role in fostering this environment. They understood the daily realities of adherence, treatment fatigue, and stigma avoidance, enabling them to offer support that felt genuine and empathetic. The guidance and validation from peers of similar age and experience helped adolescents express their concerns and explore coping strategies. Such conversations break the silence surrounding HIV, encourage mutual understanding, and provide a platform for shared problem-solving [16]. In our study, adolescents described these peer interactions as both reassuring and empowering, strengthening their sense of belonging and motivation to remain engaged in care.

### Storytelling & change

Storytelling has long been recognized as a powerful tool for patient education and behavior change. Research shows that narratives can enhance personal relevance, reduce counter-arguing, and promote the internalization of health messages [13, 16, 17]. A study with HIV-positive female adolescents in Malawi found that telling their story assisted in identifying their feelings, values, challenges, and opinions [18]. Stories provide moral lessons, offer metaphors for coping, and help individuals reframe their challenges, fostering both emotional healing and positive adjustment [19].

In our CDI study, adolescents described how story-based discussions helped them reflect on their own choices and learn from others’ experiences. This reflective process aligns with the notion of moral and emotional learning through storytelling and with evidence that supportive networks build resilience by enabling the exchange of coping strategies. During the reflection phase of the CDI group sessions, adolescents discussed what they would do differently, explored alternative actions, and collectively solved problems, illustrating how narrative engagement facilitated both emotional insight and skill development.

Peer leaders’ experiences reinforced these dynamics. As observed elsewhere, the peer-led model fostered reciprocal trust and openness because of shared lived experiences with HIV [15]. Adolescents in our study described how these shared stories reduced fear and shame, encouraged self-expression, and provided opportunities for guided reflection. This mirrors findings that reported that storytelling among HIV-positive youth in Malawi enhanced emotional understanding and self-knowledge [17].

Notably, storytelling also appeared to strengthen adherence and promote self-acceptance. Adolescents in our study frequently referenced stories that instilled ‘hope’ and demonstrated that others were ‘living successfully’ with HIV. This process of identification parallels previous findings that reauthoring personal stories can improve insight, self-efficacy, and mental health outcomes among individuals living with HIV [20]. Our participants described feeling motivated to take their medication and attend clinic appointments after hearing these narratives, illustrating how storytelling can operate simultaneously as a psychosocial and behavioral intervention.

Consistent with broader literature, adolescents also reported that storytelling made complex clinical information easier to understand than the didactic messaging typically provided by health-care. Prior studies have shown that adolescents often perceive HCWs as overly direct, unempathetic, and insufficiently skilled at discussing sensitive sexual health topics with adolescents [17, 21]. Similarly, a CDI study found that adolescents preferred seeking advice from peers rather than HCWs, sometimes leading to misinformation and risk behaviors [22]. Our findings suggest that storytelling within a peer-led framework bridges this communication gap, enabling accurate information to be conveyed in a relatable, emotionally engaging format.

All study groups in our CDI research, including adolescents, peer leaders, and HCWs described perceived short-term behavioral changes, including improved motivation to adhere to ART and attend clinic appointments. These results align with the documented benefits of age-appropriate, collaborative treatment-support strategies situated within adolescents’ lived environments and peer networks [15, 23]. Similar evidence from South Africa shows that peer-led support groups enhance adherence by enabling participants to discuss difficulties and collectively normalize their experiences of living with HIV [24].

One of the most significant changes noted in our study was an increased sense of self-acceptance of one’s HIV status. Consistent with prior findings linking non-acceptance of HIV status to poor ART adherence [25], adolescents in CDI described achieving greater acceptance and hope through hearing stories of others who were thriving. This narrative process may have underpinned the observed improvements in adherence and engagement with care.

### Challenges & sustainability

Peer leaders and HCWs offered valuable feedback on ways to strengthen and sustain the storytelling model. Both groups recommended extending peer-leader training beyond the current three days to ensure deeper comprehension of content and greater confidence in facilitating discussions. They also noted that additional sessions or longer group sessions might allow for more thorough exploration of each topic. However, previous research has cautioned that lengthier HIV interventions do not necessarily enhance behavioral outcomes, thus, careful balance is needed between depth and feasibility [26].

Peer leaders also requested additional mentoring and regular meetings with HCWs. This is consistent with findings in Zimbabwe, which showed that peer leaders in HIV programs benefit from ongoing psychosocial support to manage the emotional demands of their role [15]. Similarly, in our study, peer leaders described both pride in their responsibilities and occasional stress when handling emotionally charged discussions. Ensuring structured supervision, debriefing sessions, and access to supportive mentors could enhance both the well-being and effectiveness of peer facilitators.

Transportation and logistical barriers also affected adolescents’ participation in the support groups. Both adolescents and HCWs described difficulties attending sessions because of transport costs and long travel distances. Additional barriers included competing school and work obligations, fear of HIV status disclosure, emotional distress, and inadequate meeting spaces that limited privacy and comfort. Some HCWs also noted that adolescents who had not fully accepted or disclosed their HIV status were less likely to consistently attend sessions. These findings are consistent with broader literature documenting that ART adherence and retention among adolescents are shaped by structural, psychosocial, and stigma-related barriers, including transportation costs, school-related disruptions, disclosure concerns, mental health challenges, and unstable social environments [4, 6, 7, 25].

Overall, our findings suggest that structured peer-led storytelling groups may represent an effective, sustainable approach to promoting long-term wellness and care among adolescents living with HIV. The success of such programs depends on thoughtful story design that ensures narratives reflect relevant cultural values, communication patterns, and social structures [20]. Future adaptation and scaling should also consider cost-effective delivery methods, such as integrating mobile or web-based formats, while maintaining the social connection that underpins the intervention’s impact.

Prior studies have called for research comparing different modes of narrative delivery (e.g., video versus in-person, fictional versus third-person) and cost-effectiveness [27]. Our study supports these recommendations and highlights the importance of maintaining the interpersonal and emotional dimensions of storytelling, even as digital tools are explored. While virtual formats may enhance access, particularly given the transport challenges noted by our participants, they should be carefully designed to preserve the relational support networks that are central to the success of peer-led interventions.

#### Limitations

The study has several limitations. First, the inclusion criteria limited participation to adolescents who remained engaged in the peer-led support groups and attended at least half of the sessions. Adolescents who dropped out of the groups, refused participation, or attended fewer sessions were not interviewed, and their experiences may have provided additional insight into barriers to participation and reasons for disengagement. Because adolescents volunteered to participate in the interviews, the sample may also be biased toward individuals with more positive perceptions of the support groups.

Another important limitation relates to parental consent requirements for minors. In accordance with ethical standards, adolescents under 18 years of age were required to obtain written consent from a parent or guardian in addition to providing assent. While necessary for ethical compliance, this requirement may have unintentionally limited participation among adolescents who had not disclosed their HIV status within the family or who feared stigma associated with participation in an HIV-related program. Consequently, the perspectives of younger adolescents or those with more limited family support may be underrepresented.

In addition, all data were collected at high-volume urban health facilities in Abidjan. Adolescents receiving care in lower-volume or rural facilities may experience distinct social, structural, and health system challenges that were not captured in this study. Finally, as a qualitative study focused on participant experiences and perceptions, the findings are not intended to be generalizable to all adolescents living with HIV in Côte d’Ivoire. Nonetheless, the narratives collected from adolescents, peer leaders, and HCWs provide important insight into the perceived mechanisms, acceptability, and implementation challenges of the peer-led storytelling model within adolescent HIV care.

## Conclusion

The storytelling model provided adolescents with relatable examples that prompted discussion, reflection, and a sense of hope. Peer-led support groups created a community where adolescents could speak freely about the challenges of living with HIV, fostering trust, belonging, and mutual support. Peer leaders modeled positive behavior, and HCWs perceived improvements in adolescents’ adherence and engagement with care.

This study reinforces previous evidence on the value of peer support while offering new insights into how narrative-based interventions can facilitate comprehension, emotional connection, and self-acceptance. Strengthening peer-leader training, ensuring continued supervision, and refreshing materials will be essential for program sustainability. Further research should explore which story topics resonate most with adolescents, as well as the optimal structure and dosage of peer-leader training.

## Supplementary Information

Below is the link to the electronic supplementary material.

Supplementary Material 1

Supplementary Material 2

Supplementary Material 3

## Data Availability

The datasets used and/or analyzed for this study are available from the corresponding author on reasonable request.
