# Supplementary material for: Learning through story: how peer-led support groups use storytelling to empower adolescents living with HIV in Côte d’Ivoire
Source: BMC Health Serv Res. 2026 Jun 23;26:1246. doi: 10.1186/s12913-026-14944-x (PMC13555961; doi:10.1186/s12913-026-14944-x)
Supplement: Supplementary file 1 [file 12913_2026_14944_MOESM1_ESM.docx]

## **Evaluation of a peer-led adolescent HIV support group model in seven health facilities in Cote d’Ivoire**

## In-depth Interview with Health Care Workers

Principal Investigators: Marc Kouadio +225 22 41 45 05
Dr. Akaki Angennes +225 22 41 45 05, Dr. Leila Katirayi

Participant ID number __ __- __ __- __ __ __ (Site #/ Participant type/ individual number)

Interviewer Name: ________________________________

Site Name: ______________________________________

Interview date: __ __ / __ __ / __ __ __ __ (*dd-mm-yyyy*)

Start time: _____: ______	End time: _____ : _____

Duration of interview: _____ minutes/ _____ hours

*INSTRUCTIONS:* *This* *interview should only be started once written informed consent has been obtained from the participant. Read all of the questions and all of the information that is in bold print aloud to the participant. Use the probes as needed to gather more information from the participant. Probes should be used after the participant has spoken freely.*

**Thank you again for agreeing to participate in our study.**

**Demographic Information**

A1. Gender

Male	☐ (1)

Female	☐ (2)

A2. Which department do you work in?

Pediatric and Adolescent Unit	☐ (1)

Care and treatment Unit X	☐ (2)

Other:	☐ (3) Specify……………………………………………………………………………

A3. What is your position?

Nurse	☐ (1)

Lay Counselor	☐ (2)

Social worker	☐ (3)

Pharmacist	☐ (4)

Physician ☐ (5)

Midwife	☐ (6)

Other (specify)	☐ (7) _________________________ (write in)

A4. For how long have you been in this position at this facility?

_______ years. (For those less than one year, enter 0.)

**Working with Adolescents**

1) Please describe how your role supporting adolescents.
   Probe: Provide adolescents with drug adherence counseling, clinical appointments, psychosocial counseling, etc.
2) How do the Peer Leaders provide support to adolescents beyond the support groups?
   Probe: Providing one-on-one support to adolescents, tracking adolescents that do not show up for the support group, etc.
3) How helpful are these services provided by the Peer Leaders?
   Probe: Do these tasks affect your job? Do these tasks lessen your duties?

**Adolescent Challenges**

4) What are some of the challenges that adolescents experience when they are initiated on treatment?
   Probe: Challenges accepting their status, fear of disclosure, not wanting to take their treatment, etc.
5) Please describe the challenges that adolescents face with adherence.
   Probe: Forgetting to take their medication, challenges due to lack of disclosure, not understanding medication, not understanding drug resistance, etc.
6) Please describe the challenges that adolescents face with retention in care.
   Probe: Transport costs, challenges due to lack of disclosure, not wanting to be seen at the facility, etc.

**Effect of the Support Group**

7) How would you compare the impact of this support group on adolescents compared to the previous support groups offered?
   Probe: What differences have you seen in behavior, attitudes and general acceptance of one’s HIV status?
8) How do you think the peer-led adolescent support group changed adolescents’ behavior regarding taking their medication?
   Probe: Has medication adherence improved? Declined? Stayed the same? Why do you think this is?
9) How do you think the peer-led adolescent support group, and other services you provide to adolescents has changed adolescents’ behavior regarding attending their routine clinic appointments?
   Probe: Improved attendance? Declined attendance? Stayed the same? Why do you think this is?
10) How do you think the peer-led adolescent support group, and other services you provide to adolescents has changed adolescents’ behavior regarding their interaction with HCWs?
    Probe: Are adolescents more/less likely to listen to HCWs’ advice, recommendations, etc. How have you noticed adolescents’ attitudes towards HCWs change?

**Recommendations**

11) What can be done to improve the peer-led adolescent program?
12) Beyond the peer-led support groups, and any one-on-one support provided to adolescents, what additional support do you think adolescents need to remain adherent with their medication and retained within care?
    Probe: Please describe any additional support that you think would benefit the adolescents.

Thank the participant for their time.

Record end time at the top of the transcript.
