# Supplementary material for: Learning through story: how peer-led support groups use storytelling to empower adolescents living with HIV in Côte d’Ivoire
Source: BMC Health Serv Res. 2026 Jun 23;26:1246. doi: 10.1186/s12913-026-14944-x (PMC13555961; doi:10.1186/s12913-026-14944-x)
Supplement: Supplementary file 2 [file 12913_2026_14944_MOESM2_ESM.docx]

**Evaluation of a peer-led adolescent HIV support group model in seven health facilities in Cote d’Ivoire**

## In-depth Interview with Adolescents

Principal Investigators: Marc Kouadio +225 22 41 45 05
Dr. Akaki Angennes +225 22 41 45 05, Dr. Leila Katirayi

Participant ID number __ __ - __ - ___ ___ ___

Interviewer Name: ________________________________

Site Name: ______________________________________

Interview date: __ __ / __ __ / __ __ __ __ (*dd-mm-yyyy*)

Start time: _____: ______	End time: _____ : _____

Duration of interview: _____ minutes/ _____ hours

*INSTRUCTIONS:* *This* *interview should only be started once written informed consent has been obtained from the participant. Read all of the questions and all of the information that is in bold print aloud to the participant. Use the probes as needed to gather more information from the participant. Probes should be used after the participant has spoken freely.*

**Thank you again for agreeing to participate in our study.**

**Demographic Information**

A1. Gender

Male	☐ (1)

Female	☐ (2)

A2. Age ______________

A3. What is your marital status?

Married	☐ (1)

Single	☐ (2)

Living with partner	☐ (3)

Other	☐ (4) specify: _____________________________

A4. What is your level of education?

No school	☐ (1)

Some primary	☐ (2)

Completed primary	☐ (3)

Some secondary	☐ (4)

Completed secondary	☐ (5)

Some tertiary	☐ (6)

Completed tertiary	☐ (7)

A5. Do you have children?

No	☐ (0)

Yes	☐ (1)

A6. What month and year did you learn your HIV status? ______ month/ ______ year

A7. What month and year did you learn you start ART treatment? ______ month/ ______ year

A8. Were you previously in a support group before joining the peer-led support group?

No	☐ (0)

Yes	☐ (1)

A9. Were there any times in which you missed your ART treatment during the 6 previous months?

No	☐ (0)

Yes, less than 1 week	☐ (1)

One to 2 weeks ☐ (2)

15 days to 28 days ☐ (3)

More than 28 days	☐ (4)

***Please turn on the audio-recorder now. State the date, time and participant ID.***

**Perceptions of the peer-led adolescent support groups**

1) How do you feel about the support groups being led by adolescents?

Probe: Is this helpful to have a peer lead the group? Do you trust your peer leader? Are their challenges with having a peer lead the group? Please describe.

2) How do you feel about the ‘story-telling’ model used in the peer support group models?
   Probe: Does this model make it easier to understand messages and more applicable to your situation? Is the story usually relevant to your situation? Please tell me about any challenges with the story model.
3) How do you feel about the topics that have been selected for the support groups?
   Probe: Are the topics helpful? Are these topics that have been applicable to your life? What other topics would you like to learn more about?
4) How do you feel about the space in which the space in which the support groups meet?
   Probe: Does the space feel safe? Does the space feel private? Are you comfortable in the space? How could it be improved?
5) Did you previously attend a support group? If yes, how is the relationship with your peers different in the new adolescent model?
   Probe: More trust? Stronger friendships? More challenges?
6) What is your favorite aspect of this new peer-led model?
7) What would you most like to change about the peer-led model?
8) Did you participate in the previous support groups?
   If yes, are you more likely to attend this new model of support group compared to the previous model? Why or why not?

**Changes to ART adherence**

9) How has participating in the peer-led adolescent support group changed your understanding of HIV and how to live with HIV?
   Probe: Understand HIV better, understand the medication better, clearer understanding of how to live a positive life with HIV, etc.
10) How has participating in the peer-led adolescent support group changed your behavior regarding taking your medication?
    Probe: Miss medication less frequently? Use new techniques to remember your medication? Any advice learned from peers?
11) How has participating in the peer-led adolescent support group changed your attendance at the health facility?
    Probe: Less likely to miss clinical appointments, ensure that medication is picked up on time, follow-up with the health facility when there is a problem, etc.
12) How has participating in the peer-led adolescent support group changed how well you listen to HCWs?
    Probe: Understand the HCW messages better, more likely to communicate with the nurses when there is a challenge, no change- feel the same about HCWs, etc.

**Influence on personal life**

13) Has participating in these support groups strengthened your acceptance of your own HIV status?
    Probe: If yes, please describe how it has strengthened your acceptance. If no, have the support groups raised any additional concerns/challenges with your HIV status?
14) How has participating in the peer led groups influenced your decision to disclose your HIV status to your friends and family?
    Probe: Who have you disclosed to since joining this new peer-led model? What happened in the support group to encourage you to disclose? What advice would you give other adolescents?
15) Has participating in the peer led groups changed your relationships with your family or friends?
    Probe: Helped you to communicate better, be more understanding, etc. Raised more questions and concerns or created more challenges, etc.

**Recommendations**

16) What recommendations do you have to improve the peer-led adolescent support groups?
    Probe: Changes to 10 topics, changes with the story-telling model, changes with the peer leaders, etc.
17) What additional support do you think you need to strengthen your ability to take your medicine routinely and always return to the facility?
    Probe: What would help you? Please suggest any ideas, it does not need to be related to the support group.

Thank the participant for their time.

Record end time at the top of the transcript
