# Supplementary material for: Learning through story: how peer-led support groups use storytelling to empower adolescents living with HIV in Côte d’Ivoire
Source: BMC Health Serv Res. 2026 Jun 23;26:1246. doi: 10.1186/s12913-026-14944-x (PMC13555961; doi:10.1186/s12913-026-14944-x)
Supplement: Supplementary file 3 [file 12913_2026_14944_MOESM3_ESM.docx]

## **Evaluation of a peer-led adolescent HIV support group model in seven health facilities in Cote d’Ivoire**

## In-depth Interview with Peer Leaders

Principal Investigators : Dr Marc N’goran +225 22 41 45 05
Dr. Akaki Angennes +225 22 41 45 05, Dr. Leila Katirayi

Participant ID number __ __ - __ - ___ ___ ___

Interviewer Name: ________________________________

Site Name: ______________________________________

Interview date: __ __ / __ __ / __ __ __ __ (*dd-mm-yyyy*)

Start time: _____: ______	End time: _____ : _____

Duration of interview: _____ minutes/ _____ hours

*INSTRUCTIONS:* *This* *interview should only be started once written informed consent has been obtained from the participant. Read all of the questions and all of the information that is in bold print aloud to the participant. Use the probes as needed to gather more information from the participant. Probes should be used after the participant has spoken freely.*

**Thank you again for agreeing to participate in our study.**

**Demographic Information**

A1. Gender

Male	☐ (1)

Female	☐ (2)

A2. Age ______________

A3. What is your marital status?

Married	☐ (1)

Single	☐ (2)

Separate	☐ (3)

Divorced	☐ (4)

Widowed	☐ (5)

Living with partner	☐ (6)

A4. What is your level of education?

No school	☐ (1)

Some primary	☐ (2)

Completed primary	☐ (3)

Some secondary	☐ (4)

Completed secondary	☐ (5)

Some tertiary	☐ (6)

Completed tertiary	☐ (7)

A5. How many children do you have? ___________

A6. Child has been on RAL for:

One week	☐ (1)

Four weeks	☐ (2

***Please turn on the audio-recorder now. State the date, time and participant ID.***

**Structure of the peer-led Adolescent Support Group**

1) Did you participate or facilitate the previous support groups?
   Probe: How does the new support group compare to the previous models?
2) How well do you think the story-telling model works?
   Probe: What are the benefits? What are challenges?
3) How appropriate are the ten selected topics?
   Probe: Do these topics meet the needs of the adolescents? What additional topics would be helpful?

**Ability to lead the peer-led support groups**

4) Please describe the training you received to be a Peer Leader.
   Probe: Did you feel that the training was adequate? Have you encountered any challenges where you felt you were not prepared
   Probe: Did you receive training for the topics, using the story telling methods, leading the group, etc.
5) What additional information would you have liked to receive?
6) Do you feel like you are able to answer the peers’ questions when they arise?
   Probe: What are some of the questions that may arise that are outside of your knowledge?
   Probe: What do you do when a topic is asked outside of your knowledge area?
7) Please describe any additional roles you have to support the adolescents outside of the peer-led adolescent groups
   Probe: Assisting adolescents at the facility, following up with adolescents that have missed a support group, having one-on-one meetings with adolescents outside the support group, support to reach a adolescent treatment goal, etc. Please probe to see what other roles they may have.
8) How feasible is your role as a peer leader?
   Probe: Is the workload acceptable? Is the work enjoyable? Do you anticipate that you would like to continue in this role for some time?

**Adolescent challenges with treatment**

9) What were some of the main challenges adolescents’ experienced remaining adherent on care?
   Probe: not accepted their HIV status yet, side effects of medication, not understanding the need to take medicine routinely/developing resistance, challenges with taking medication when have not disclosed, etc.
10) What were some of the main challenges adolescents’ experienced remaining in care and attending their routine clinic appointments
    Probe: cost of travel to the facility, challenges traveling to the facility when they have not disclosed their HIV status, challenges with the HCWs, etc.
11) For any adolescents that dropped out/missed the peer-led support group meetings, what were their reasons?
    Probe: what were the reasons given when you followed up with these adolescents?

**Impact of the peer-led support groups on adolescents HIV care**

12) How do you think the peer-led adolescent support group, and other services you provide to adolescents has helped adolescents accept their HIV status?
    Probe: Please describe anything said or done by the adolescents to indicate that the services have helped adolescents with accepting their HIV status.
13) How do you think the peer-led adolescent support group, and other services you provide to adolescents has changed adolescents’ behavior regarding taking their medication?
    Probe: What changes do adolescents discuss in regards to taking their medication?
14) How do you think the peer-led adolescent support group, and other services you provide to adolescents has changed adolescents’ behavior regarding attending their routine clinic appointments?
    Probe: What changes do adolescents discuss in regards to attending their clinic appointments. Why do you think this is?
15) How do you think the peer-led adolescent support group, and other services you provide to adolescents has changed adolescents’ behavior regarding their interaction with HCWs?
    Probe: How have you noticed adolescents’ attitudes towards HCWs change?
16) How do you think the peer-led adolescent support group, and other services you provide to adolescents has improved adolescents ability to live their life positively with HIV?
    Probe: Please describe any changes in adolescent attitudes, behavior, perspectives, etc.

**Recommendations**

17) What can be done to improve the peer-led adolescent program?
    Probe: Change the structure of the peer-led adolescent support groups? Change the topics of the information provided? Improve the space in which the support groups meet, include parenting session, etc.
18) Beyond the peer-led support groups, and any one-on-one support provided to adolescents, what additional support do you think adolescents need to remain adherent with their medication and retained within care?
    Probe: Please describe any additional support that you think would benefit the adolescents.

Thank the participant for their time.

Record end time at the top of the transcript
